# Prevalence of Metabolic Syndrome and Framingham Risk Score in Vegetarian and Omnivorous Apparently Healthy Men

**DOI:** 10.5935/abc.20180083

**Published:** 2018-05

**Authors:** Francisco Antonio Helfenstein Fonseca, Maria Cristina de Oliveira Izar

**Affiliations:** Escola Paulista de Medicina, Universidade Federal de São Paulo (UNIFESP), São Paulo, SP - Brazil

**Keywords:** Metabolic Syndrome, Diet, Vegetarian, Healthy Lifestyle, Prevention and Control

The Life Style Heart Trial^1^ was a milestone; published in 1990, the study showed that a healthy lifestyle associated with a vegetarian diet can promote the regression of coronary lesions, even in patients not using lipid-lowering drugs.

There is a large epidemic of cardiovascular disease today. Sedentary lifestyle, obesity, diabetes and dyslipidemia are triggers of mechanisms related to cardiovascular diseases, such as changes in the gut microbiota, increase in inflammatory markers and prothrombotic factors, and impaired immune response.

Traditional guidelines provide information on the identification of patients at high risk, and establishment of therapy targets of cardiovascular disease in advanced stage when only lifestyle changes seem insufficient for an effective, early reduction in cardiovascular events.

In 1988, Gerald Reaven delivered a historical Banting Lecture,^2^ presenting a link between insulin resistance and obesity, hyperglycemia, hypertension and dyslipidemia (notably hypertriglyceridemia and low HDL-c levels). Reaven was the pioneer in describing the Metabolic Syndrome, initially named X Syndrome, which constituted a high-risk situation for coronary disease. The author showed that high cholesterol levels are not the only mechanism for this condition, but rather, other components of the syndrome could be deeply modified by lifestyle changes.^3^^-^^5^

In the present issue of *Arquivos Brasileiros de Cardiologia*,^6^ the authors show that vegetarian diet is strongly associated with a lower prevalence of Metabolic Syndrome and a lower cardiovascular risk predicted by the Framingham score as compared with an omnivorous diet, defined by individuals who consumed at least four portions of meat a week.

The study included a relatively small, but homogeneous sample of apparently healthy adults. In addition, the total number of male subjects was higher than that in the classical study by Ornish (The Lifestyle Heart Trial). Its cross-sectional design limits the ability to make any inferences, which should be confirmed by prospective studies. However, the strict inclusion criteria (minimum of four years following a vegetarian or omnivorous diet) and the quality of the results obtained from the subjects demonstrate the importance of nutrition and how different metabolic, clinical and laboratory aspects are between these groups.^6^

Recently, during the annual American College of Cardiology’s meeting (ACC.18), professor Valentin Fuster suggested different strategies for primoradial (first 25 years of age), primary (25-50 years) and secondary (after 50 years of age) prevention, aiming at reducing the incidence and complications of cardiovascular diseases.

Results of this study on vegetarian diet highlight not only its effects on individual markers of cardiovascular risk, but also substantial changes in the global risk score and Metabolic Syndrome components.^6^

We have witnessed a phase of epidemiological transition, where the greatest challenges are not to achieve cholesterol, glycemic and blood pressure control, but to primarily reduce obesity and metabolic disorders associated with hyperglycemia. Much attention has been paid to patients with coronary, cerebrovascular or renal events or to advanced peripheral vascular disease. However, a greater impact on the health of a larger number of people would be achieved if we focused on simpler, lifestyle changing measures, towards primordial and primary prevention.
